# HIV-1-Associated Left Ventricular Cardiac Dysfunction in Humanized Mice

**DOI:** 10.1038/s41598-020-65943-9

**Published:** 2020-06-16

**Authors:** Prasanta K. Dash, Fadhel A. Alomar, Bryan T. Hackfort, Hang Su, Amy Conaway, Larisa Y Poluektova, Howard E. Gendelman, Santhi Gorantla, Keshore R. Bidasee

**Affiliations:** 1 Department of Pharmacology and Experimental Neuroscience, University of Nebraska Medical Center, Omaha, NE 68198 USA; 2 Department of Cellular and Integrative Physiology, University of Nebraska Medical Center, Omaha, NE 68198 USA; 3 Department of Environment and Occupational Health, University of Nebraska Medical Center, Omaha, NE 68198 USA; 4 Department of Pharmacology and Toxicology, College of Clinical Pharmacy, Imam Abdulrahman Bin Faisal University, Dammam, Kingdom of Saudi Arabia; 5 Nebraska Redox Biology Center, Lincoln, NE USA

**Keywords:** HIV infections, Cardiovascular biology, Cardiovascular diseases

## Abstract

The molecular cause(s) for early onset heart failure in people living with HIV-1 infection (PLWH) remains poorly defined. Herein, longitudinal echocardiography was used to assess whether NOD.Cg-Prkdc^scid^ Il2rgt^m1Wjl^/SzJ mice reconstituted with human hematopoietic stem cells (Hu-NSG mice) and infected with HIV-1_ADA_ can recapitulate the salient features of this progressive human disease. Four weeks post infection, Hu-NSG mice of both sexes developed left ventricular (LV) diastolic dysfunction (DD), with 25% exhibiting grade III/IV restrictive DD with mitral regurgitation. Increases in global longitudinal and circumferential strains and declines in LV ejection fraction and fractional shortening were observed eight weeks post infection. After twelve weeks of infection, 33% of Hu-NSG mice exhibited LV dyskinesia and dyssynchrony. Histopathological analyses of hearts seventeen weeks post infection revealed coronary microvascular leakage, fibrosis and immune cell infiltration into the myocardium. These data show for the first time that HIV-1_ADA_-infected Hu-NSG mice can recapitulate key left ventricular cardiac deficits and pathophysiological changes reported in humans with progressive HIV-1 infection. The results also suggest that HIV-1 infected Hu-NSG mice may be a useful model to screen for pharmacological agents to blunt LV dysfunction and associated pathophysiologic causes reported in PLWH.

## Introduction

Advances in and adherence to combination anti-retroviral therapies (cART) have resulted in significant increases in life expectancy for people living with HIV-1 infection (PLWH)^1^. A consequence of the increased longevity however is an increase in the prevalence of cardiovascular diseases (CVD) including heart failure (HF) for which the mechanisms remain poorly defined and pharmacologic strategies to attenuate them are limited^2,3^ also from (https://www.hiv.gov/hiv-basics/overview/data-and-trends/global-statistics). At present, nearly 38 million people worldwide are infected with the human immunodeficiency virus type one (HIV-1)^4^, with ~1.1 million of them living in the United States^5^ also from (https://www.hiv.gov/hiv-basics/overview/data-and-trends/global-statistics). By the year 2030, studies estimate that 73% of these individuals will be >50 years of age, and >25% of them will develop impairments in cardiac function leading to HF^2–4^. This symptomatic HF will also begin up to a decade earlier in PLWH compared to the general population^5^. Although the underlying cause(s) for impairments in cardiac functions in PLWH remain poorly understood, clinical data suggest that chronic inflammation associated with long-term cART usage, co-infections, alcohol and drugs of abuse may be promoting/potentiating metabolic dysregulation and aging-related diseases in this population^2,3,5,6^.

Prior to the wide spread use of cART, PLWH commonly developed AIDS-defining cardiomyopathies. Autopsied as well as echocardiographic studies done in the early 1980’s revealed both right and left ventricular hypertrophy/dilations as well as defects in mitral and tricuspid valves^7–10^. Others also reported Kaposi’s sarcoma (primary-ascending aorta, sub-epicardial adipose tissue and metastatic), lymphomas, non-bacterial thrombolytic endocarditis, fibrinous pericarditis and atrophy in autopsied hearts from HIV-1 infected individuals^11,12^. Later studies showed that the opportunistic co-infections including *Pneumocystis, Tuberculosis, Toxoplasma, Cytomegalovirus, Cryptococcus, Aspergillus, Nocardia, Candida, Strongyloidiasis, Zygomycetes and Herpes* were likely contributing to the pathobiology and/or severity of HF development in HIV-1 infected patients^13^. In addition to opportunistic infections, azidothymidine (zidovudine), the principal antiretroviral agent used at that time also contributed to the development of HF in HIV-1 infected individuals^14,15^.

The introduction of cART in 1995 significantly lowered peripheral viral load, and the incidence of AIDS-defining systolic HF dramatically declined^16^ also from (https://www.hiv.gov/hiv-basics/overview/data-and-trends/global-statistics). However, the prevalence of LV diastolic dysfunction with myocardial ischemia and fibrosis persisted^17–20^. These diastolic defects are also occurring at younger ages, with rates among women and children higher than in men^21,22^. Long-term use of cART, persistent low-grade inflammation, HIV-1 auxiliary proteins, alcohol, and drug of abuse have been identified as likely causes for LV diastolic dysfunction in PLWH in the cART era^23–26^. However, the extent to which these factors contribute to and pharmacological strategies to attenuate LV dysfunction in the setting of HIV-1 infection, remain limited in part due to paucity of relevant animal models that can support productive replication of the human-specific HIV-1 virus.

To date, transgenic rodent models have successfully delineated the contributions of the HIV-1 auxiliary proteins, Nef, gp120 and Tat in the development of HIV-1 associated HF^24,27–31^. However, since these models do not support productive replication of this human-specific virus, the impact of antiretrovirals and inflammation in the setting of HIV-1 infection remains inadequately addressed. Simian immunodeficiency virus (SIV) infected macaques provided an excellent non-human primate model for studying HIV pathogenesis^32–34^. SIV is closely related to HIV on a genetic level, and mimics human AIDS in many important aspects . However, the SIV model also has major disadvantages, as they are expensive and must be housed in accredited primate facilities. A small animal model replicating HIV pathogenesis would facilitate the studies on mechanisms involved in the development and progression of HF and to test the therapeutic interventions.

Earlier, we showed that humanized mouse models reconstituted with human hematolymphoid system can be productively infected with HIV-1. We also showed that these models can mirror pathological features as seen in patients, including HIV replication, CD4+ T cell depletion, lymphadenopathy, and immune activation^35–46^. Herein, longitudinal echocardiography was used to assess whether NOD.Cg-Prkdc^scid^ Il2rgt^m1Wjl^/SzJ (NSG) mice reconstituted with human (Hu) hematopoietic stem cells (HSC) and infected with HIV-1 can recapitulate the left ventricular dysfunction reported with HIV-1 infection. There is an urgent need for rodent models that not only support productive replication of HIV-1 but can also recapitulate the salient features of this progressive cardiovascular disease in humans. We also conducted histopathological analyses on *ex vivo* hearts from HIV-1-infected Hu-NSG mice to determine if impairment in microvascular permeability and fibrosis are contributing to the cardiac pump failure during progressive HIV-1 infection.

## Results

### Characterization of humanized mice used in study

Twenty-four Hu-NSG mice (also termed as humanized mice) of both sexes reconstituted at birth with human hematopoietic stem cells and infected with HIV-1_ADA_ after twenty weeks were used. At 4, 8, 12- and 16-weeks post-infection, multiple modality echocardiography (Pulse wave, M-mode, Tissue doppler and speckle tracking) were conducted. Blood samples were also collected to assay viral load, CD4+ and CD8+ T cell count.

Human CD45+ immune cell reconstitution ranged from 15 to 60% of peripheral blood white blood cells (Supplemental Table 1). HIV-1 infection led to productive infection, with plasma HIV-1 viral loads peaking at eight weeks post infection (wpi, 1.3 ± 0.2 × 10^6^ RNA copies/ml), and declining to 1.3 ± 0.2 × 10^5^ RNA copies/ml at 17 weeks (Table 1). The viral loads of infected mice were provided in Supplemental Table 1. The percentages of CD4+ T cells in blood declined in HIV-1 infected Hu-NSG mice from 76.6 ± 0.4 to 37.2% ± 2.1 while CD8+ T cells gradually increased over the same period (Supplemental Fig. 1). The animals maintained their body masses to within 5% of their starting weights (Table 1). Echocardiographic analyses failed to identify significant differences in left ventricular mass between Hu-NSG infected and uninfected mice throughout the study (Table 1). However, when hearts were excised and weighed at the end of the study, mean heart weight of HIV-1 infected Hu-NSG mice was significantly lower than that of aged-matched uninfected controls (Table 1).

**Table 1:** Evaluations of animals used in study.

	Parameters	At start, t = 0	4 weeks	8 weeks	12 weeks	17 weeks
Hu-NSG mice (n = 8)	Body weight (g)	18.3 ±0.4	18.0 ±0.9	18.8 ±0.8	18.9 ± 1.0	19.2 ±0.6
	Left ventricular mass (mg)	60.6 ± 1.9	62.4 ± 4.5	62.0 ± 3.9	60.3 ± 5.3	62.6 ± 7.3
	Heart weight (mg)	NA	NA	NA	NA	155.2 ± 2.2
	% CD4+ T cell in blood	76.6 ± 0.4	77.4 ± 1.1	78.1 ± 0.7	77.2 ± 0.6	75.2 ± 0.5
Hu-mice infected with HIV-1_ADA_ (n = 16)	Body weight (g)	18.3 ± 0.4	19.0 ± 0.5	19.4 ± 0.5*	19.3 ± 0.6	18.8 ± 0.6
	Left ventricular mass (mg)	60.6 ± 1.9	61.7 ± 5.7	62.4. ± 4.1	58.3 ± 5.6	57.9 ± 5.3
	Heart weight (g)	NA	NA	NA	NA	142.8 ± 3.1**
	HIV-1 plasma viral load (RNA copies/ml)	0	1.2 ± 0.1 × 10^5^	1.3 ± 0.2 × 10^6^	1.5 ± 0.2 × 10^5^	1.4 ± 0.3 × 10^5^
	% CD4+ T cell in blood^@^	76.6 ± 0.4	60.8 ± 1.5*	50.1 ± 1.3*	42.1 ± 1.6*	37.2 ± 2.1*

NA- not applicable.

*Significantly different (p < 0.05) from start.

**Significantly different (p < 0.05) from Hu-mice not infected.

^@^gating strategy-CD45→CD3→CD4.

### Echocardiographic analyses

Echocardiography is a non-invasive technique that provides quantitative information on the dimensions, contractile kinetics, as well as tissue and blood velocities in the heart^47^. Speckle tracking (ST) also captures segmental tissue motion of the heart in multiple planes and axes over the cardiac cycle and provided detailed information on myocardial strain^48–50^.

**Figure 1:**
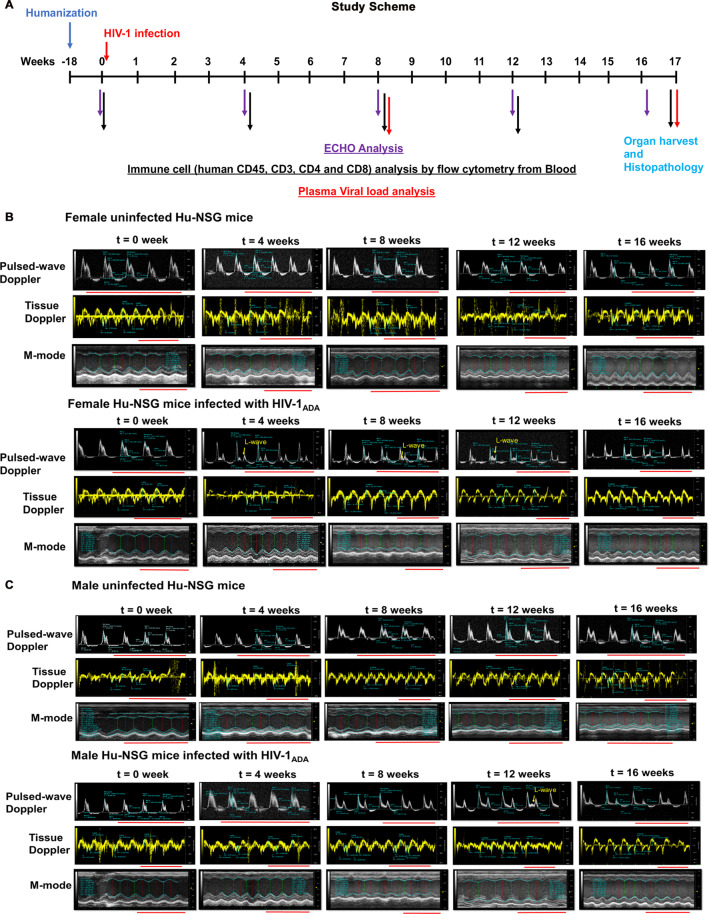
Longitudinal left-ventricular echocardiographic images from uninfected and HIV-1 infected Hu-NSG mice. (**A**) Shows the schematic of the experimental design of the study starting from reconstitution of new born mice with human CD34+ human hematopoietic stem cells, HIV-1 infection, viral and immune profiling, echocardiographic analysis and histopathological evaluations. **(B,C)** Representative longitudinal echocardiographic images in a female and a male Hu-NSG mouse performed prior to (t = 0 weeks) and at 4, 8, 12 and 16 weeks after injection with saline or infection with HIV-1 using pulse-wave Doppler to assess diastolic function (filling mechanisms), and tissue Doppler to assess tissue velocity and M-mode imaging to assess ventricular contractility. In this study, the onset of diastolic dysfunction began earlier in females than in males. Red line below each image represents 0.5 sec.

**Figure 2:**
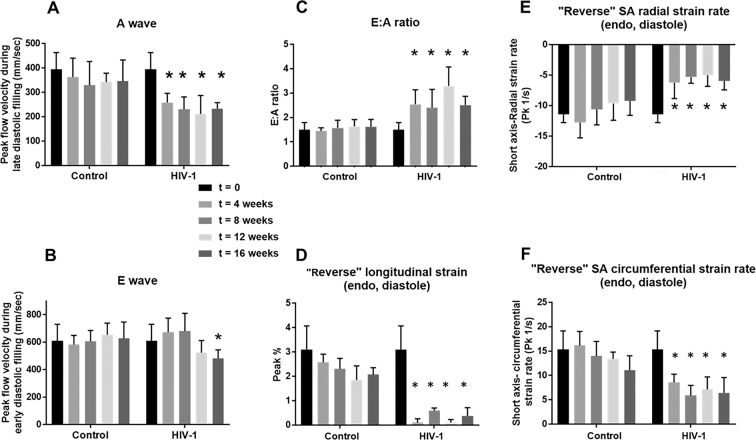
Longitudinal changes in key diastolic parameters and “reversed strain” parameters over a 16-week period after HIV-1 infection. Panel (A) shows mean late transmitral velocity (A), Panel (B**)** shows early transmitral velocity (E), and Panel (C) show E: A ratios of peak early- and late-diastolic transmitral velocities measured every four weeks over a 16-week period using Pulsed-wave Doppler echocardiography. Panels **(**D–F) show reversed longitudinal strain, and radial and circumferential strain rates during early LV filling obtained from analyses of B-mode images obtained every four weeks over the 16-week period. For these measurements, the “reverse peak” algorithm in the Vevo Strain Software was utilized to analyze the B-mode images. Data shown are mean ± SEM for n = 8 uninfected Hu-NSG mice (3 male and 5 females) and n = 16 for HIV-1 infected Hu-NSG mice (4 male and 12 females). * denote significantly different from t = 0 weeks (p < 0.05) within the group.

Four weeks following HIV-1 infection, all Hu-NSG mice (4 males and 12 females) developed varying degrees of LV diastolic dysfunction (DD)^49,50^, also Supplemental Fig. 2C. One male mouse developed degree I; Two males and 4 females developed pseudo-normal, degree II; and 1 male and 8 females developed degree III-IV DD (Supplemental Fig. 2C). Figure 1A shows the experimental time line describing the pre and post time points of HIV-1 infection and immune, viral and echocardiographic analysis. Figure 1B,C shows representative longitudinal pulsed-wave, tissue Doppler and M-mode parasternal short-axis echocardiographic images of a female and a male Hu-NSG mouse prior to (t = 0) and 4, 8, 12 and 16 weeks after saline injection or HIV-1 infection. Four of eight female Hu-NSG mice with degree III-IV also exhibited L-waves in their pulsed-wave Doppler images (Fig. 1B, yellow arrows), indicative of mitral regurgitation^13,51,52^. Degrees III-IV also known as restrictive filling disease, arose from a reduction in peak late-diastolic transmitral velocity, A-wave (Fig. 2A) and not the peak early-diastolic transmitral velocity, E-wave (Fig. 2B), resulting in mean E:A ratios were >2.5 (Fig. 2C). Heart rates of infected Hu-NSG mice were not significantly different from uninfected controls (Supplemental Fig. 3H).

**Figure 3:**
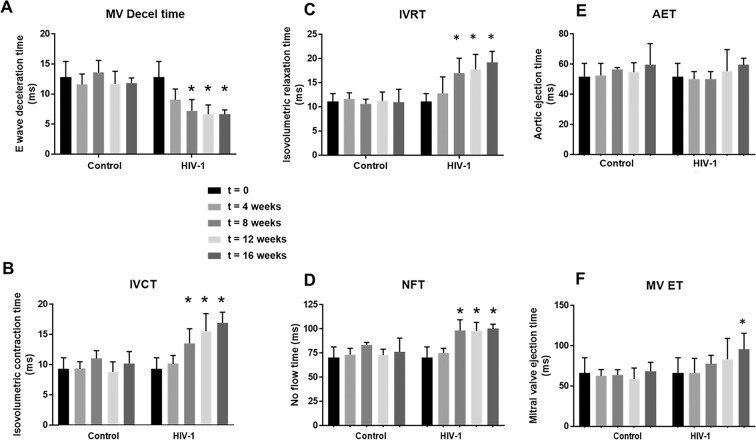
Longitudinal changes in additional diastolic parameters over a 16-week period post-HIV-1 infection. Panels (A–F**)** shows mean E-wave deceleration times, IVCT, IVRT, NFT, AET, and MV-ET in Hu-NSG mice prior to (t = 0), and 4, 8, 12, and 16 weeks after injection with saline or infection with HIV-1. Data shown are mean ± SEM for n = 8 uninfected Hu-NSG mice (3 males and 5 females) and n = 16 for HIV-1 infected Hu-NSG mice (4 males and 12 females). * denote significantly different from t = 0 weeks (p < 0.05) within the group.

Other pulsed-wave Doppler parameters including E-wave deceleration time, isovolumetric relaxation (IVRT), isovolumetric contraction (IVCT), mitral valve ejection time (MV-ET), aortic ejection time (AET), and no flow times (NFT) remained unchanged after 4 weeks of HIV-1 infection (Fig. 3). However, ST analyses of B-mode images using the “reverse peak algorithm”^53^ revealed a significant increase (p < 0.05) in longitudinal strain (Pk, %) and decreases in radial and circumferential strain rates (Pk, 1/s), consistent with increased left ventricular stiffening (Fig. 2D–F), an underlying cause of DD.

**Figure 4:**
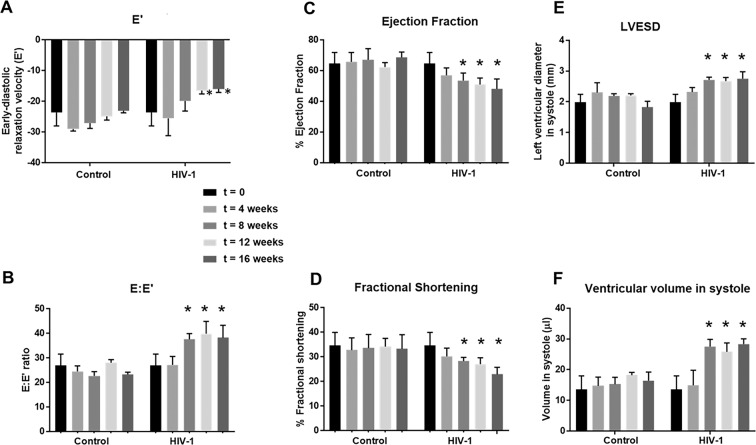
Longitudinal changes in systolic parameters and tissue relaxation velocity in HIV-1 infected Hu-NSG and Control mice. Panels (A–D**)** show changes in ejection fraction, fractional shortening, left ventricular end diastolic diameter and the amount of blood in the heart at the end of contraction (left ventricular end diastolic volume) in Hu-NSG mice prior to (t = 0), and 4, 8, 12, and 16 weeks after injection with saline or infection with HIV-1. Panels (E,F) shows early-diastolic relaxation velocity (E′), and (**B**) shows the ratio of early diastolic transmitral flow velocity to early-diastolic relaxation velocity (E:E′) in Hu-NSG mice prior to (t = 0), and 4, 8, 12, and 16 weeks after injection with saline or infection with HIV-1. Data shown are mean ± SEM for n = 8 uninfected Hu-NSG mice (3 males and 5 females) and n = 16 for HIV-1 infected Hu-NSG mice (4 males and 12 females). * denote significantly different from t = 0 weeks (p < 0.05) within the group.

**Figure 5:**
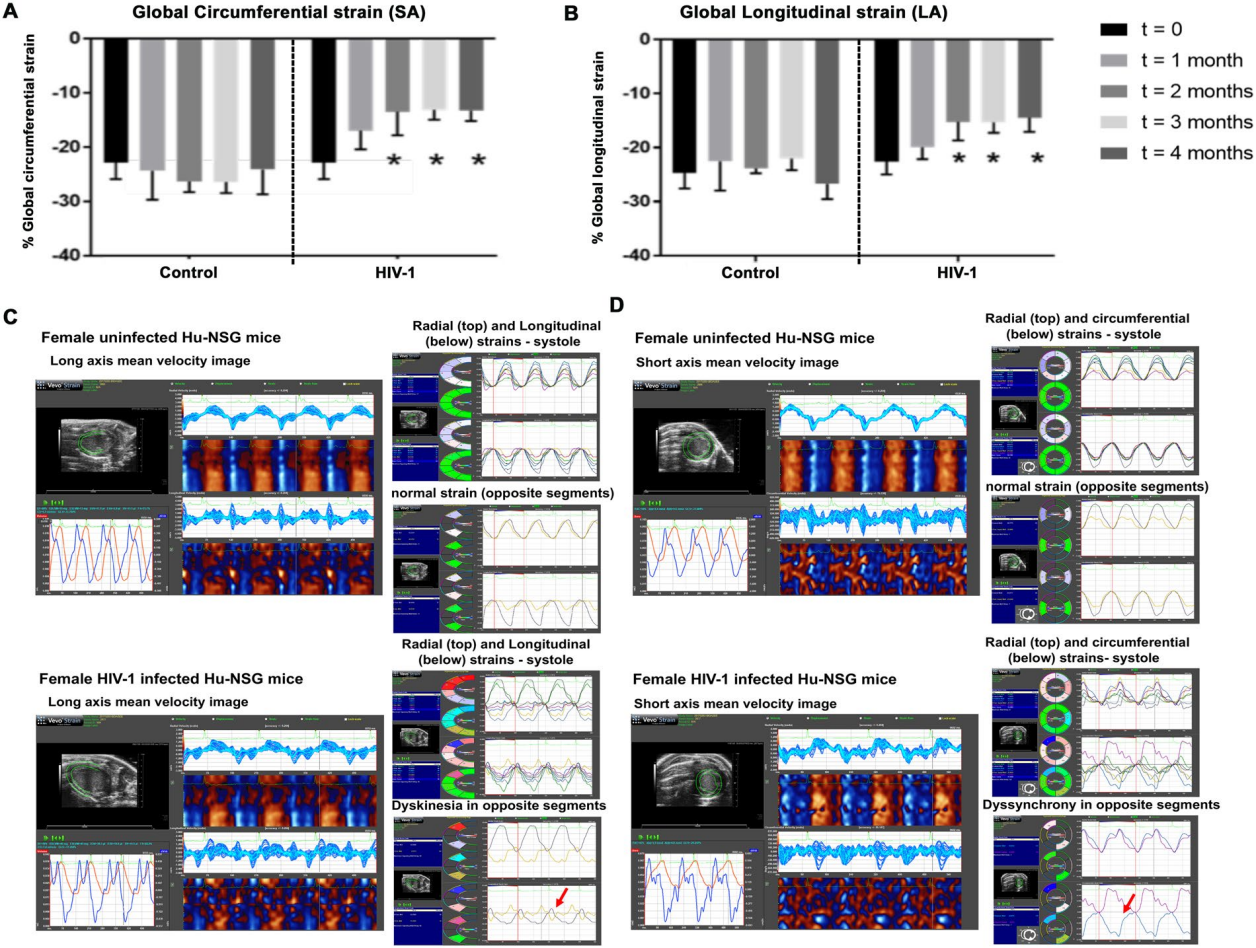
Changes in global longitudinal and circumferential strains over a 16-week period post-HIV-1 infection in Hu-NSG Mice. (**A,B**) Shows mean global longitudinal and circumferential strains in Hu-NSG mice prior to (t = 0), and 4, 8, 12, and 16 weeks after injection with saline or infection with HIV-1. Data shown are mean ± SEM for n = 8 uninfected Hu-NSG mice (3 males and 5 females) and n = 16 for HIV-1 infected Hu-NSG mice (4 males and 12 females). * denote significantly different from t = 0 weeks (p < 0.05) within the group. (**C,D**) Shows long and short axes velocities during three to four consecutive cardiac cycles (left), and radial and longitudinal strain in six and two opposite segments (right) from an uninfected female and a HIV-1-infected female Hu-NSG mouse that exhibits dyskinesia (expansion of a wall segment during systole, (**C**). right panel, red arrow) and dyssynchrony (opposite walls moving in counter directions), (**D**), right panel, red arrow after 12 weeks of infection. Images in 5C and 5D are representative of n = 6 uninfected and HIV-1 infected Hu-NSG mice (4 females and 2 males). White scale bar at the bottom indicates 200 μm.

After four weeks of infection, M-mode Doppler recordings did not show any significant changes in left ventricular function (percent fractional shortening (FS), percent ejection fraction (EF), stroke volume, cardiac output, and dimensions (anterior wall thickness- diastole (LVAW;d); anterior wall thickness-systolic (LVAW;s), posterior wall thickness-diastolic (LVPW;d) and posterior wall thickness-systolic (LVPW;s)) and LV mass, in male and female mice (Fig. 4A,B and Supplemental Fig. 3A–G). ST analyses of B-mode images using the “normal peak algorithm” (systole) also did not show any significant differences in global longitudinal and circumferential strains (Fig. 5A,B) or in six segment (anterior base, AB; anterior middle, AM; anterior apex, AP, posterior base, PB; posterior middle, PM; and posterior apex PA) analyses of circumferential, longitudinal, radial strain/strain rates (data not shown). Tissue Doppler also did not reveal any significant changes in mean early-diastolic tissue relaxation velocity, (E′) (Fig. 4A,B).

After eight weeks of infection, the majority of females (11/12) and half of the males (2/4) HIV-1 infected mice developed degree III-IV diastolic impairments. The other HIV-1-infected Hu-NSG mice (2 males and 1 female) exhibited pseudo-normal, degree II DD. L-waves persisted in the four female Hu-NSG mice infected with HIV-1, but these waves were less pronounced compared to that seen in four weeks infection (Fig. 1B,C, yellow arrows). E-wave deceleration time decreased (Fig. 3A) and IVRT, IVCT and NFT increased significantly (Fig. 3B–D). Mean AET and MV-ET remained unchanged (Fig. 3E,F). Mean early-diastolic tissue relaxation velocity (E′) also did not change significantly (Fig. 4D), but since E-wave velocity decline (Fig. 2C), mean E:E′ increased (Fig. 4F). The increase in reversed longitudinal strain (Pk, %) and decreases in radial strain rate (Pk, 1/s) and circumferential strain rate (Pk, 1/s) during diastole also persisted (Fig. 2D–F). After eight weeks of infection, there were small but significant decreases in EF and FS (Fig. 4A,B), and increases in LVESD and left ventricular end systolic volume (LVESV, Fig. 4E,F). Mean stroke volume, cardiac output, LVAW;d, LVAW;s, LVPW;d, LVPW;s and LV mass remained unchanged (Supplemental Fig. 3). ST analyses of B-mode images during systole, also showed significant increases in global longitudinal and circumferential strains (Fig. 5A,B). However, mean radial and circumferential strain and strain rates during systole (six-segment analyses) did not change (Fig. S4). None of the Hu-NSG mice showed significant impairment in LV diastolic or systolic functions eight weeks after injection with saline (control). There were also no significant differences in LV diastolic and systolic parameters between males and females uninfected Hu-NSG mice.

After twelve weeks of infection, 15/16 HIV-1-infected Hu-NSG mice (3 males and 12 females) developed degree III-IV DD with significant declines in E′ (Fig. 4A). L-waves were also pronounced in two male Hu-NSG mice  infected with HIV-1 (Fig. 1C, yellow arrow lower right panel) after 12 weeks of infection. FS and EF declined further (Fig. 4A,B) and LVESD and LVESV increased further (Fig. 4C,D). Global longitudinal and circumferential strains also increased (Fig. 5A,B). Video recordings of parasternal long- and short-axis loops showing direction and magnitude of endocardial deformation between uninfected (control) and HIV-1 infected Hu-NSG mice are shown in Supplemental Videos 1–4. One female HIV-1 infected Hu-NSG mice also exhibited pericardial effusion (data not shown). ST analyses of long (Fig. 5C) and short axes B-mode (Fig. 5D) images during systole also revealed dyskinesis (expansion of a wall segment during systole Fig. 5C,D) and dyssynchrony (opposite walls moving in counter directions, Fig. 5C,D) in 6/16 Hu-NSG mice (4 females and 2 males). These six HIV-1 infected Hu-NSG mice also exhibited significant increases in longitudinal strain maximum time-to-peak (T2P) delay between the earliest and the latest segment (T2P) delay (42.3 ± 5.2 ms after t = 12 weeks of infection compared to 31.1 ± 2.2 ms at t = 0 weeks, p < 0.05), time-to-peak variation, defined as the standard deviation (STD) of T2P over all six segments (17.1 ± 2.2 ms at t = 12 week compared to 11.1 ± 1.2 ms at t = 0 weeks, p < 0.05) and STD of [T2P/RR interval] for each segment RR interval [0.14 ± 0.01 at 12 week compared to 0.10 ± 0.01 ms at t = 0 weeks, p < 0.05). Left ventricular dyskenesis and dyssynchrony persisted in the 6/16 Hu-NSG mice assayed at sixteen weeks of infection. LV systolic and diastolic dysfunctions also worsened. None of the Hu-NSG mice injected with saline showed LV dysfunction up to 16 weeks.

### Microvascular leakage, macrophage infiltration and fibrosis

Dysregulation of endothelial cells (ECs) is a contributing cause for LV cardiac dysfunction in progressive HIV-1 infection^54^. When ECs lose their ability to dilate microvessels, pressure will build up within the vasculature, promoting transcytosis of substances from the blood into the myocardium. Impairment in the function and a decrease in expression of tight junction proteins will also promote the movement of substances from the blood into the interstitium (leakiness)^55^. Increased transcytosis/leakiness will also promote inflammation and tissue fibrosis.

**Figure 6:**
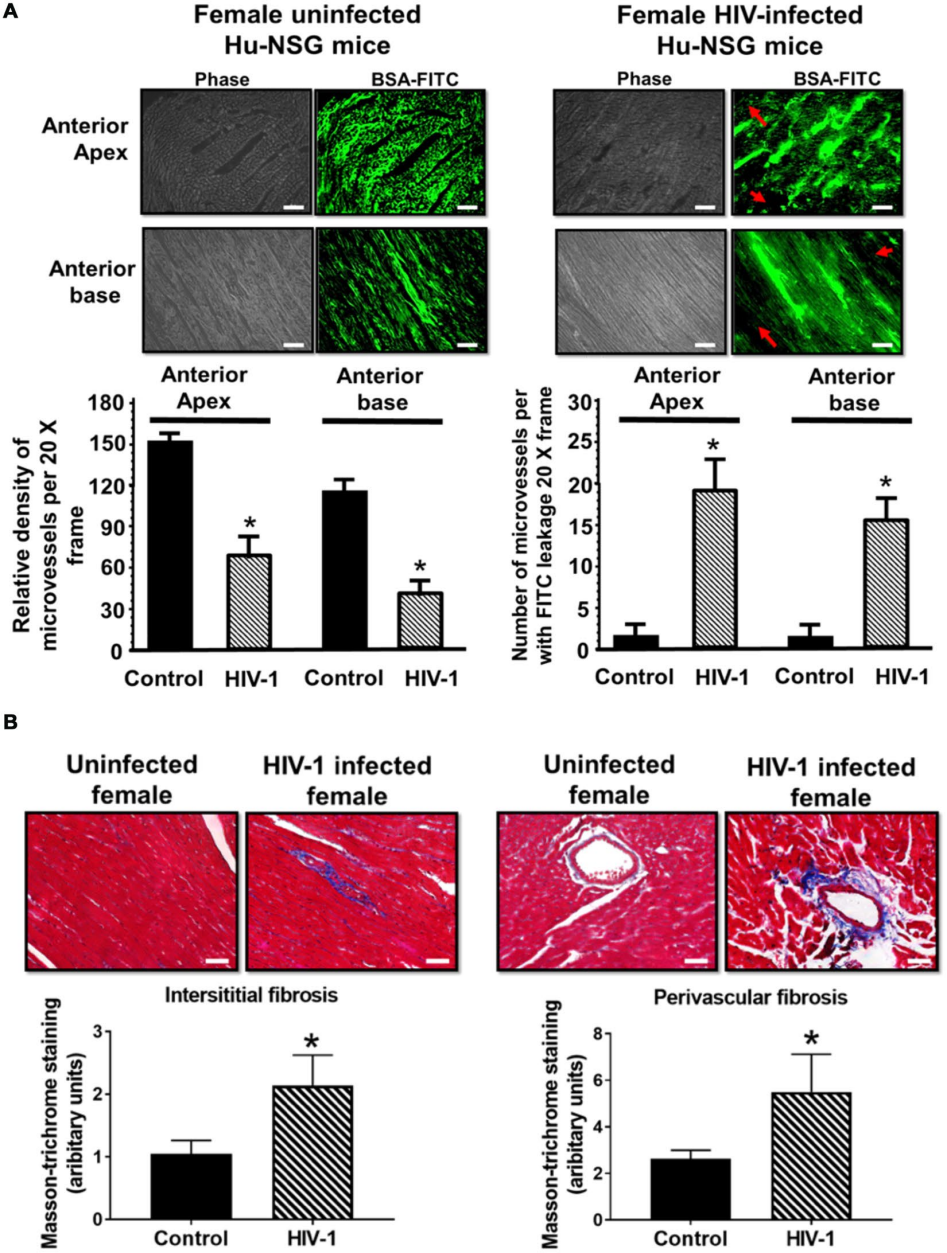
Microvessel perfusion, immune cell and fibrosis in ventricular tissues in HIV-1 infected humanized and control mice. **(A)** left shows representative images of BSA-FITC from the anterior apex and anterior base from Hu-NSG mice seventeen weeks after injection with saline or infected with HIV-1. Red arrows point to ischemic regions in which there are no microvessels perfused with BSA-FITC. Graphs below show relative density of microvessels (<25 µm) perfused with BSA-FITC per 20-x frame. Values are mean ± SEM from n > 20 sections from n = 6 mice, (4 females and 2 males). * denote significantly different (p < 0.05) compared to saline injected humanized mice. Panel (B) shows Masson Trichrome staining for fibrosis in LV sections from Hu-NSG mice seventeen weeks after injection with saline and infected with HIV-1. Graphs below are mean ± SEM from n > 20 sections from n = 6 mice (4 females and 2 males). * denote significantly different (p < 0.05) compared to saline injected humanized mice. White scale bar at the bottom indicates 200 μm.

One week after the last echocardiography (17 weeks), BSA-FITC was intravenously injected into Hu-NSG mice with or without HIV-1 infection to assess coronary microvascular permeability. In left ventricular tissues from uninfected Hu-NSG mice, the green fluorescence of BSA-FITC was confined to the microvascular networks, indicative of minimal leakage of substances from the blood into the myocardium. The green BSA-FITC fluorescence was also widely distributed throughout the tissue, indicative of extensive perfusion of microvessels. Representative images from LV anterior apex and base are shown in Fig. 6A, left panel. Interestingly, in left ventricular tissues from HIV-1 infected Hu-mice, the green BSA-FITC fluorescence was confined to the microvasculature; in some areas “green blobs” were seen, indicative of leakage of BSA-FITC into the interstitium. The density of microvessels perfused with BSA-FITC per 20x frame was also significantly reduced in infected Hu-NSG mice, possibly a consequence of upstream microvascular leakage. Representative images from anterior apex and base are shown in Fig. 6A, right upper panels with mean data shown in the graphs below.

An increase in microvascular permeability is expected to promote infiltration of immune cells into the myocardium. Immunohistological staining of paraffin embedded heart tissues revealed significant increase in the number of HLA-DR+ human leukocytes in heart tissues from HIV-1 infected humanized mice compared to uninfected controls (Supplemental Fig. 5). Most of the cells were CD68+ macrophages and cells positive for HIV-1p24 protein expressions were rarely seen. Increased number of human leukocytes were seen both at 4- and 12-weeks post-infection. Tissues from a separate set of mice sacrificed at specified time points after infection were used for analysis (data not shown). HIV-1 viral load in peripheral blood of these mice ranged from 10^4^ to 10^6^ viral RNA copies/ml.

Studies have reported increased fibrosis in hearts of HIV-1-infected individuals^20^. Since coronary microvascular leakage can promote fibrosis, we investigate whether fibrosis is also increased in hearts of Hu-NSG mice. Figure 6B (upper) shows representative light microscopy photomicrographs of Masson’s Trichrome stained images from hearts of female control and infected humanized mice, emphasizing significant increase in extracellular matrix deposition (fibrosis) in interstitial (left images)and perivascular region (right images) compared to aged-matched controls. Mean data are presented in graphs.

## Discussion

The principal finding of the present study is that HIV-1-infected Hu-NSG mice develop progressive LV diastolic and systolic HF, akin to that reported with HIV infection reported pre-cART^56^era. This cardiomyopathy starts with impairment in LV diastolic function (defects in cardiac filling mechanisms) that progressively worsens to systolic dysfunctions (defects in pumping mechanism) with left ventricular dyskinesis and dyssynchrony (increases the risk of arrhythmias)^10,13,56–59^. The decline in ejection fraction in Hu-NSG mice after 16 weeks of infection was ~25% (from 64.6 ± 7.2% at t = 0 weeks to 48.1 ± 6.1% at t = 16 weeks), indicating that the LV systolic dysfunction developed with HIV-1 only was not as severe as that reported in patients with co-infections in the pre-cART era. Whether longer duration of infection will result in dilated cardiomyopathy remains to be determined.

Although LV diastolic dysfunctions (DD) was prominent in both males and females as early as four weeks of infection, the severity varied between males and female mice, with 8/12 females exhibiting degree III-IV DD (restrictive DD) and 1/4 male exhibiting degree III-IV DD. These data are consistent with literature reports indicating that females with HIV-1 infection are at a higher risk of developing HF than their male counterparts^60,61^. To date, the specific reason(s) for the more aggressive DD phenotype in female HIV-1 infected Hu-NSG mice remain poorly understood, but may be related to sex hormones, and possibly increased susceptibility to systemic amyloidosis, sarcoidosis, and eosinophil infiltration into the heart^62,63^. The A-wave in pulsed-wave Doppler is generated from atrial contraction and a smaller and shorter duration A-wave arises from a “stiffer” LV. An increase in inflammation will trigger endothelial cell dysfunction, microvascular leakage, macrophage infiltration, fibrosis and impairment in intracellular Ca^2+^ handling, contributing causes for LV stiffening^64^.

In this study, L-waves were detected in 4/8 female HIV+ Hu-NSG mice after four weeks of infection, and in 2/4 HIV-1 infected male Hu-NSG mice after 12 weeks of infection, a phenomenon that has been reported in HIV-infected individuals in pre and post cART eras^13,51^. It should also be noted that the mean heart rate of HIV-1-infected Hu-NSG mice with L-waves were similar to infected Hu-NSG mice without L-wave, emphasizing that the separation of E and A waves were not from slowing of heart rate (see Supplemental Fig. 3H). The L-wave in pulsed wave Doppler arises when pulmonary vein mid diastolic flow through the left atrium and into the LV across mitral valve continues after early rapid filling^52^. It is also indicative of mitral regurgitation that arises from dysfunctional mitral valves^52^. Mitral regurgitation is a contributing cause of pulmonary hypertension, a well-known co-morbidity in PLWH^65,66^. Whether HIV-1 infected Hu-NSG mice with L-waves are developing pulmonary hypertension and associated right ventricular dysfunction is not known at this time. The latter is especially intriguing, since if it holds true, this subpopulation of infected Hu-NSG mice could also serve as a new model to investigate underlying causes and treatment strategies to attenuate pulmonary hypertension in the context of HIV-infection. Additional work is needed to determine if right ventricular (RV) dysfunction also develops in these HIV-1-infected mice that exhibit L-waves.

As the duration of infection increased, DD worsened in both male and female HIV-1 Hu-NSG mice. The HIV-1 infected male Hu-NSG mouse that developed degree I (delayed relaxation) at four weeks progressed to degree II (pseudo-normal), and those with degree II progressed to degree III-IV (restrictive) after eight weeks of infection. Pulse wave echocardiography also revealed worsening in the filling mechanisms as indicated by an increase in E-wave deceleration time and an increase in IVRT, changes that have been reported in HIV-infected individuals^51,59^. The E wave is dictated by the left atrial pressure during early diastole and in restrictive cardiac disease, the faster E-wave deceleration time (or rapid cessation of flow) results either from an increase in left ventricular pressure or more rapid emptying of the LA into a very compliant LV. Since hearts of HIV-1 infected Hu-NSG mice became stiffer, we attributed the faster deceleration time to an increase in LV pressure^64^. IVRT is the time after the aortic valve closes and the mitral valves open. Relaxation of the heart during this phase of diastole is energy dependent, requiring ATP to transport Ca^2+^ released from troponin C into the sarcoplasmic reticulum (SR) by sarco(endo)plasmic reticulum Ca^2+^-ATPase (SERCA2)^67^. An increase in IVRT arises when the release of Ca^2+^ from troponin C is impaired, SERCA2’s ability to transport Ca^2+^ from the cytoplasm into the SR becomes compromised, or both.

After eight weeks of infection, echocardiography revealed small but significant decreases in FS and EF, and increases in global longitudinal and circumferential strains, consistent with reduced contractility (a systolic defect). IVCT also increased, indicative of an impairment in the initial phase of LV contraction (early systole). The specific mechanism(s) underlying the reduction in contractility and increase in IVCT remain undefined, but we posit that increases in longitudinal and circumferential strains arising from hypertrophy and/or fibrosis could be contributing causes. Defects in evoked Ca^2+^-induced Ca^2+^ release (CICR) may also be another cause^68^.

After 12 weeks of viral infection, dyskinesia and dyssynchrony were also observed in males and female Hu-NSG mice (4 females and 2 males). It is well known that left ventricular dyskinesia and dyssynchrony increase the risk of arrhythmias, maladies previously reported in HIV-infected patients^69–71^. Implantable defibrillator and cardiac resynchronization are the mainstay of treatments for cardiac dyssynchrony, but there is some hesitance to the use these devices in HIV-1 infected patients for fear of other types of infections due to their compromised immune function^72^. Delineating the causes and identifying strategies to attenuate left ventricular dyskinesia and dyssynchrony in the settings of HIV-1 infection could be an important step forward in attenuating these life-threatening conditions in HIV-1 patients.

The second major finding of the present study is that microvascular permeability increases in hearts of HIV-1 infected Hu-NSG mice. This increase in vascular permeability of BSA-FITC suggest that substances as large as 65 kDa can traverse from the blood into the myocardium, via smaller-diameter microvessels (capillaries and arterioles, <25 µm). Why some but not all microvessels become “leaky”, remains poorly understood at this time. What we know thus far is that HIV auxiliary proteins, inflammatory cytokines and metabolites can decrease expression of tight junction proteins on microvascular endothelial cells^54,73^. Loss of tight junction proteins on microvascular endothelial cells will potentiate the movement of cells (monocytes/macrophages, leukocytes and eosinophils) and proteins from the blood into the myocardium triggering inflammation. How soon after infection microvascular leakage starts remains undefined.

The third major finding of the present study is that interstitial and perivascular fibrosis are significantly increased in hearts of HIV-1 infected Hu-NSG mice, accounting for the increased myocardial stiffness. Although specific mechanisms for the increased myocardial fibrosis is not clear at this time, transcytosis of substances as well as immune cells from the blood into the cardiac interstitium will trigger inflammation^74^ activate matrix metalloproteinases (MMP’s) and increase deposition of collagen fibers^75^. An increase in myocardial inflammation will also activate the inflammation-induced transcription factor NF-κB (p-P65) to increase expression of an array of inflammation-induced proteins in hearts of Hu-NSG mice. Macrophage infiltration was also evident in the hearts of HIV-1 infected mice. Increased soluble CD163 and macrophage inflammation with cardiac fibrosis in post-mortem heart tissues from HIV infected humans and in SIV infected macaques was shown^76^. Additional work will be needed to define the specific proteins that are altered in hearts of infected Hu-NSG mice.

In conclusion, the present study shows for the first time that HIV-1 infected Hu-NSG mice (both males and females) develop LV cardiac dysfunction in longitudinal manner, akin to that reported in people with progressive HIV-1 infection (see Supplemental Table 2). The defect starts with an impairment in LV filling mechanisms (diastolic defects) and progressively worsens to impairments in LV contractions, dyskinesia and dyssynchrony. Female Hu-NSG infected with HIV-1 develop more severe diastolic defects than their male counterparts, but specific mechanisms for this remain poorly defined. About 25% of the cohort of the female HIV-1 infected Hu-NSG mice also developed mitral regurgitation, an underlying cause for pulmonary hypertension and right ventricular dysfunction. Although LV systolic dysfunction occurred in all HIV-1 infected mice, mean EF after sixteen weeks of infection (equivalent to 24 years of infection in humans) was ~48%, suggestive of heart failure with preserved ejection fraction (HFpEF). These data are consistent with the development of a less severe cardiomyopathy in the absence of co-infections^13^. At the end of the sixteen-week, hearts of HIV-1 infected Hu-NSG mice exhibited significant coronary microvascular leakage, immune cell infiltration into the myocardium, and fibrosis, possible causes for the increased LV stiffness, and LV diastolic and systolic dysfunctions. Whether intracellular Ca^2+^ cycling mechanisms become compromised in hearts of HIV-1 infected Hu-NSG mice remain poorly defined. From these new data sets we also posit that HIV-1 infected Hu-NSG can serve as a novel model to delineate pathophysiologic mechanisms that contribute to, and for identifying novel pharmacological agents to blunt HIV-associated cardiomyopathy.

## Materials and Methods

### Antibodies and reagents

Human hematopoietic stem cell enrichment was done using magnetic beads conjugated CD34+ antibodies from Miltenyi Biotec Inc., Auburn, CA, USA. Primary antibodies used for human immune cell reconstitution in mice by flow cytometry and immunohistochemistry were to human antigens CD45, CD19, CD14, CD8, CD4, and CD3 (BD Pharmingen, San Diego, CA, USA) and they were fluorescence conjugated. Human CD68 primary antibodies were also obtained from BD Pharmingen, San Diego, CA, USA. Goat anti-human IgG coupled to horse radish peroxidase secondary antibodies (Cat # 62–8420) were obtained from Thermo-Fisher Scientific, Grand Island NY). Bovine serum albumin labeled with fluorescein isothiocyanate (BSA-FITC, Cat# A9771) and Trichrome (Masson) staining kit (Cat# HT15-1KT) were from Sigma-Aldrich (St Louis, MO, USA). All other reagents used were of the highest grade commercially available.

For flow cytometric analysis we used a panel of antibodies comprised of FITC-conjugated mouse anti-human CD45, Alexa Fluor 700-conjugated mouse anti-human CD3, APC-conjugated mouse anti-human CD4, and BV421-conjugated mouse anti-human CD8, PE-conjugated mouse anti-human CD14 and PE-Cy5-conjugated mouse antihuman CD19 antibodies as a six-color combination to measure human pan-CD45, CD3, CD4, CD8, CD14 and CD19 positive cell reconstitution. Flow cytometric analysis was performed using LSR-II FACS analyzer (BD Biosciences, Mountain View, CA, USA).

### Ethics statement

All experimental protocols involving the use of laboratory animals were approved by the University of Nebraska Medical Center (UNMC) Institutional Animal Care and Use Committee (IACUC) ensuring the ethical care and use of laboratory animals in experimental research. All animal studies were performed in compliance with UNMC institutional policies and NIH guidelines for laboratory animal housing and care. Human CD34+ cells were isolated from umbilical cord blood obtained from UNMC labor and delivery department at UNMC with written consents from adult parents to use the remaining or discarded biological material for research. Samples were collected without identifiers under UNMC Institutional Review Board (IRB) exempt. The UNMC institutional IRB determined that these studies using anonymized cord blood samples doesn’t constitute human subject research as defined at 45CFR46.102(f). We regularly collect the cord blood samples and isolate CD34+ hematopoietic cells which are either injected immediately into mice or stored in liquid nitrogen for future human reconstitution. NOD.Cg-*Prkdc*^*scid*^*Il2rg*^*tm1Wjl*^/SzJ (NSG) mice were obtained from the Jackson Laboratories (Bar Harbor, Maine, USA; stock number 005557), and a breeding colony was developed at the University of Nebraska Medical Center. All animal procedures are approved under the University of Nebraska Medical Center IACUC protocols 18-110-08 and 10-107-01 for ECHO procedures. There are no other human biological samples being used in the current study.

### Generation of Hu-NSG (humanized) mice

Humanized NSG (Hu-NSG) mice were generated as described earlier^35–37^. For this, new born NSG mice were irradiated with a sub-lethal dose of radiation (1 Gy) using a RS‐2000 X‐Ray Irradiator (Rad Source Technologies). CD34+ Hu-NSG were enriched from human cord blood using immune-magnetic beads (CD34+ selection kit; Miltenyi Biotec Inc., Auburn, CA, USA). CD34+ cell purity was >90% as confirmed by flow cytometry and then injected intra-hepatically (IH) at 50,000 cells/mouse. Each donor derived cells were used to reconstitute from 2 to 10 mice depending on the sample size and yield. Mice were bled at subsequent time points from the submandibular vein into ethylenediaminetetraacetic acid (EDTA)-coated tubes and screened for human immune cells using flow cytometry. For flow cytometric analysis we used a panel of antibodies comprised of FITC-conjugated mouse anti-human CD45, Alexa Fluor 700-conjugated mouse anti-human CD3, APC-conjugated mouse anti-human CD4, and BV421-conjugated mouse anti-human CD8, PE-conjugated mouse anti-human CD14 and PE-Cy5-conjugated mouse antihuman CD19 antibodies as a six-color combination to measure human pan-CD45, CD3, CD4, CD8, CD14 and CD19 positive cell reconstitution. Flow cytometric analysis was performed using LSR-II FACS analyzer (BD Biosciences, Mountain View, CA, USA). Antibodies and isotype controls were obtained from BD Pharmingen, San Diego, CA, USA, and staining was analyzed with a FlowJo (BD Immunocytometry Systems, Mountain View, CA, USA). Results were expressed as percentages of total number of gated lymphocytes. The gating strategy for immune cell analysis for the whole study at all the time points has now been provided in Supplemental Fig. 6. The percentages of CD4 and CD8 positive cells were obtained from human CD3 + gate. Humanization of the animals was affirmed by flow cytometry with a range of CD45 percentage from 15–60. Around 5% of hu-HSC mice develop graft versus-host disease (GVHD), and mice with signs of GVHD were eliminated from the study. Data in this study were generated from twenty-four humanized mice and their details including the sex, CD34+ Hu-NSG donor and human CD45+ cell reconstitution at 20 weeks age is given in Supplemental Table 1.

### HIV-1 infection

Sixteen humanized mice (Hu-mice, 4 males and 12 females) were infected intraperitoneally (IP) with 2 × 10^4^ tissue culture infectious dose 50 (TCID_50_) of HIV-1_ADA_ at 20 weeks age^35–39,46^. Eight Hu- NSG mice served as uninfected aged-matched controls. Peripheral blood samples were collected every four weeks via submandibular vein bleeding to assess HIV-1 viral RNA. Plasma HIV-1 RNA levels were measured using an automated COBAS Ampliprep V2.0/Taqman-48 system (Roche Molecular Diagnostics, Basel, Switzerland) as per the manufacturer’s instructions. The detection limit after dilution factor adjustment was 400 viral RNA copies/ml.

### Conventional echocardiography

Transthoracic conventional echocardiography was performed using a Fujifilm VisualSonics Vevo 2100 system (Fujifilm VisualSonics, Toronto, ON, CAN) employing a MS550D transducer with a center frequency of 40 Hz and an axial resolution of 40 µM, prior to and 4, 8, 12, and 16 weeks after infection with HIV-1 or saline injection. For this, hair on chests of mice were removed (Nair, Church & Dwight Co., Inc. NJ, USA). Twenty-four hours later, mice were anesthetized with 1–3% isoflurane (Cardinal Health, Dublin OH, USA) and taped in the supine position on a heated 37 °C pad. Anesthesia was maintained with 0.5–3% isoflurane via a nose cone. Feet of mice were connected to ECG leads, and pulsed-wave Doppler images were acquired in the parasternal short axis mode with appropriate stage tilt and probe tilt to acquire maximum flow (Supplemental Fig. S2A, upper) and digitally stored in cine loops. The offline Program Vevo LAB 3.1.1 was then used to assess peak early- and late-diastolic transmitral velocities (E and A waves), E-wave deceleration time, isovolumetric relaxation time (IVRT), isovolumetric contraction time (IVCT), mitral valve ejection time (MV ET), aortic ejection time (AET), and no flow time (NFT) as indices of diastolic function/dysfunction (Supplemental Fig. S2B,C). E/A ratio was also calculated. M-mode images were acquired from parasternal short-and long axes views to assess left ventricular (LV) end-diastolic diameter (LVEDD), LV end-systolic diameter (LVESD), LV anterior wall thickness- diastole (LVAW;d), LV anterior wall thickness-systolic (LVAW;s), LV posterior wall thickness-diastolic (LVPW;d) and LV posterior wall thickness-systolic (LVPW;s), LV mass, fractional shortening (FS), and percent ejection fraction (EF) (Supplemental Fig. S2A, and 2D). Early-diastolic tissue relaxation velocity (E′) was measured using Tissue doppler, and E/E′ ratio was calculated. Analyses were done in a blinded manner but decoded for statistical evaluation.

### Speckle tracking echocardiography

Parasternal long- and short-axes B-mode images were obtained at a rate of >300 frames/second using the Fuji VisualSonics Vevo 2100 system (Fujifilm VisualSonics, Toronto, ON, CAN) and digitally stored in cine loops. Vevo LAB 3.1.1 was used to determine global longitudinal and circumferential strain using three to four consecutive cardiac cycles (Supplemental Fig. 2E, left). The Vevo Strain Software was used to determine circumferential, longitudinal, radial strain/strain rates, and dyskinesis during systole using six segment (anterior base, AB; anterior middle, AM; anterior apex, AP, posterior base, PB; posterior middle, PM; and posterior apex PA) analyses (Fig. S2E, right). The “reverse peak” algorithm which coincides with early LV filling during diastole was also used to assess early ventricular stiffness^53^. All analyses were done in a blinded manner, but decoded for statistical analyses. LV dyssynchrony was determined from longitudinal strain using three different methods: (a) maximum time-to-peak (T2P) delay between the earliest and the latest segment, (b) time-to-peak variation, defined as the standard deviation (STD) of T2P over all six segments, (c) STD of [T2P/RR interval] for each segment RR interval was obtained with Vevo Strain Software. Analyses were done in a blinded manner but decoded for statistical evaluation.

### Microvessel perfusion and permeability

One week after the last echocardiographic measurement (17 weeks after infection), mice were injected with bovine serum albumin coupled to fluorescein isothiocyanate (BSA-FITC, 40 mg/kg in sterile 1X PBS buffer, 50 µL) via a tail vein^55^. BSA-FITC was allowed to circulate for 10 min, after which animals were anesthetized with 5% isoflurane chest cavities were opened and hearts were quickly removed and immersed in 4% paraformaldehyde (PFA) for 24 hrs at 4 °C. Hearts were then transferred to 4% PFA/15% sucrose solution for 24 hrs, and then 30% sucrose solution for 24 hrs. Cryoprotected hearts were cut into 20 μm thick longitudinal/coronal sections on a microtome (Leica EM-UC 6, Leica Microsystems, Wien, Austria) and mounted onto pre-cleaned glass slides. Cardiac sections were then washed three times with 1X PBS to remove cutting medium. Vectashield mounting medium containing DAPI was added to the sections, and slides were cover slipped and dried overnight. Next day slides were placed on the head stage of a Nikon TE2000 microscope attached to a Coolsnap HQ2 CCD camera (Photometrics, Tuscon AZ, USA) and images were collected to assess the density of microvessels perfused with BSA-FITC and microvascular leakage. For determining the density of perfused microvessels, 20X frames from three adjacent sections were analyzed. To be counted as a perfused vessel, the vessel must contain BSA-FITC (green) in a length ≥20 µm. Branched vessels were counted as one. A vessel was counted as leaky when BSA-FITC was seen emanating from the confines of its walls. Analyses were done in a blinded manner but decoded for statistical evaluation.

### Immune cell infiltration

Cardiac tissues from Hu-NSG mice with or  without HIV-1 infection were embedded in paraffin as described earlier. Five micrometer sections were then cut and placed onto glass slides. Slides were de-paraffinized with xylene (3 changes, ten minutes each) and rehydrated in decreasing concentrations of ethanol (100%, 95%, 70% and distilled water, three minutes each) followed by phosphate-buffered saline wash. Immuno-histochemical assay was then used to determine T-lymphocytes (HLA-DR and CD68+ human macrophages in hearts of uninfected and HIV-1 infected Hu-NSG mice. Horse serum (10%) was used as the blocking agents to reduce non-specific interactions. Primary and secondary antibody concentrations were 1:100 to 1:200, respectively. Diaminobenzidine (DAB) was used as the visualizing agent. Images were taken with a Nikon inverted fluorescence microscope (TE 2000). Nikon Elements image analysis software was used to quantitation.

### Fibrosis

Cardiac tissues from Hu-NSG mice with or without HIV-1 infection were embedded in paraffin as described earlier. Five micrometer sections were then cut and placed onto glass slides. Slides were de-paraffinized with xylene (3 changes, ten minutes each) and rehydrated in decreasing concentrations of ethanol (100%, 95%, 70%, 50% and distilled water, three minutes each) followed by phosphate-buffered saline wash. Fibrosis was assessed using the Masson Trichrome staining kit as per manufacturer’s instructions without modification (Sigma-Aldrich, St Loius, MO, USA). Sections were then dehydrated using increasing concentrations of ethanol (80, 95 and 100%) and three changes of Xylene incubations for 5 mins in each solution and then cover slipped with Prolong Gold Anti-fade reagent. Images were then taken with a Nikon inverted fluorescence microscope (TE 2000) equipped with a CoolSNAP HQ2 CCD Camera (Photometrics, Tucson, AZ, USA). Nikon image analysis software was then used to quantitate changes in a blinded manner. Analyses were done in a blinded manner but decoded for statistical evaluation.

### Statistical analyses

Data were analyzed using GraphPad Prism 7.0 software (La Jolla, CA) and presented in text as the mean $\pm$ the standard error of the mean. All experiments listed in this manuscript were performed using a minimum of three biologically distinct replicates. Sample sizes were not based on power analyses as the magnitude of cardiovascular deficits was not known nor could it be projected. For comparisons of two groups, Student’s *t* test (two-tailed) was used. Viral loads were analyzed by one-way ANOVA with Bonferroni correction for multiple-comparisons. For studies with multiple time points, two-way factorial ANOVA and Bonferroni’s post-hoc tests for multiple comparisons were performed. Animal studies included a minimum of five animals per group. Extreme outliers beyond the 99% confidence interval of the mean and 3-fold greater than the SEM were excluded. Significant differences were determined at *p* < 0.05.

## Supplementary information

Supplementary information.

Supplementary information 2.

Supplementary information 3.

Supplementary information 4.

Supplementary information 5.

## Data Availability

Data are available from the corresponding author upon reasonable request.
