# Supplementary material for: HIV-1-Associated Left Ventricular Cardiac Dysfunction in Humanized Mice
Source: Sci Rep. 2020 Jun 16;10:9746. doi: 10.1038/s41598-020-65943-9 (PMC7297773; doi:10.1038/s41598-020-65943-9)
Supplement: Supplementary file 1 — Supplementary information. [file 41598_2020_65943_MOESM1_ESM.pdf]

HIV-1-Associated Left Ventricular Cardiac Deficits in Humanized Mice

SUPPLEMENTAL MATERIALS

Prasanta K Dash[1#], Fadhel A. Alomar[4#], Bryan T. Hackfort[2], Hang Su[1], Amy Conaway[1], Larisa Poluektova[1], Howard E. Gendelman[1], Santhi Gorantla[1*] and Keshore R. Bidasee[1, 3, 5*]

Departments of [1]Pharmacology and Experimental Neuroscience, [2]Cellular and Integrative Physiology, and [3]Environment and Occupational Health, University of Nebraska Medical Center, Omaha, NE 68198, [4]Department of Pharmacology, College of Clinical Pharmacy, Imam Abdulrahman bin Faisal University, Kingdom of Saudi Arabia and [5]Nebraska Redox Biology Center, Lincoln NE.

# Equal Contributions made

*** Corresponding authors**

Santhi Gorantla, Ph.D. (Shared correspondence)

Department of Pharmacology and Experimental Neuroscience, 985800 Nebraska Medical Center, Durham Research Center, DRC 8020, Tel: (402) 559-8754; Fax: (402) 559-7495, email: sgorantla@unmc.edu

Keshore R. Bidasee, Ph.D. (Manuscript Submission, Shared correspondence)

Department of Pharmacology and Experimental Neuroscience, 985800 Nebraska Medical Center, Durham Research Center, DRC 3047, Omaha, NE, USA. Tel: (402) 559-9018; Fax: (402) 559-7495, email: kbidasee@unmc.edu

Supplemental Table 1: Characteristics of Humanized mice used in the study

Mice Numbers	Gender	Cord blood sample*	% Human CD45+ cells in peripheral blood	Experimental group	Plasma Viral load (HIV-1 RNA copies/ml)
2823	F	A	14.4	Control	-
2825	F	A	23.7	Control	-
2841	F	A	21.4	Control	-
2842	F	A	16.4	HIV	45200
2860	M	B	54.9	HIV	42600
2866	F	B	48.8	Control	-
2867	F	B	39.6	HIV	209000
2868	F	B	52.7	HIV	8000
2869	F	B	44.4	HIV	209000
2870	F	C	28.5	HIV	60200
2872	F	C	32.7	HIV	204000
2873	F	C	31.8	HIV	434000
2874	F	C	27.8	HIV	210000
2875	M	C	27.8	HIV	109000
2876	M	C	22.2	Control	-
2877	M	C	29.3	HIV	293000
3769	F	D	54.0	HIV	262000
3795	F	E	36.1	Control	-
3797	M	E	38.2	Control	-
3801	M	E	60.0	Control	-
3762	F	F	52.7	HIV	25000
3766	F	F	54.8	HIV	26900
3772	F	G	62.6	HIV	101000
3774	M	G	51.0	HIV	27600

*Each cord blood sample is used to reconstitute 2 to 6 mice.

Supplemental Figures with Legends

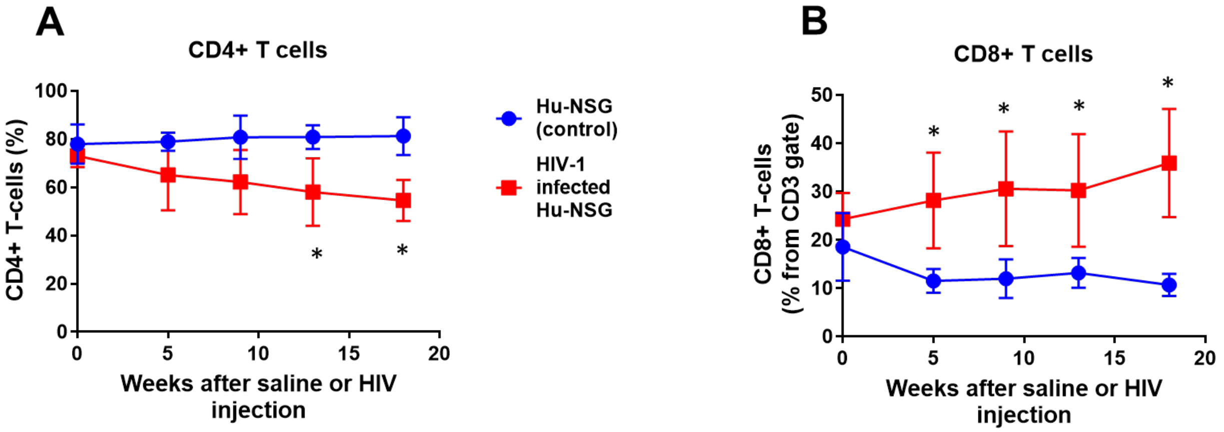

Supplemental Fig. 1: Time-frame of observation of immune status of animals used in the study. Graphs A and B show longitudinal CD4+ and CD8+ cell populations in peripheral blood from uninfected Hu-NSG mice and HIV-1-infected Hu-NSG mice. Data shown are mean ± SEM for n = 8 uninfected Hu-NSG mice (3 males and 5 females) and n = 16 for HIV-1 infected Hu-NSG mice (4 males and 12 females). * denote significantly different from t = 0 weeks ($p<0.05$) within the group.

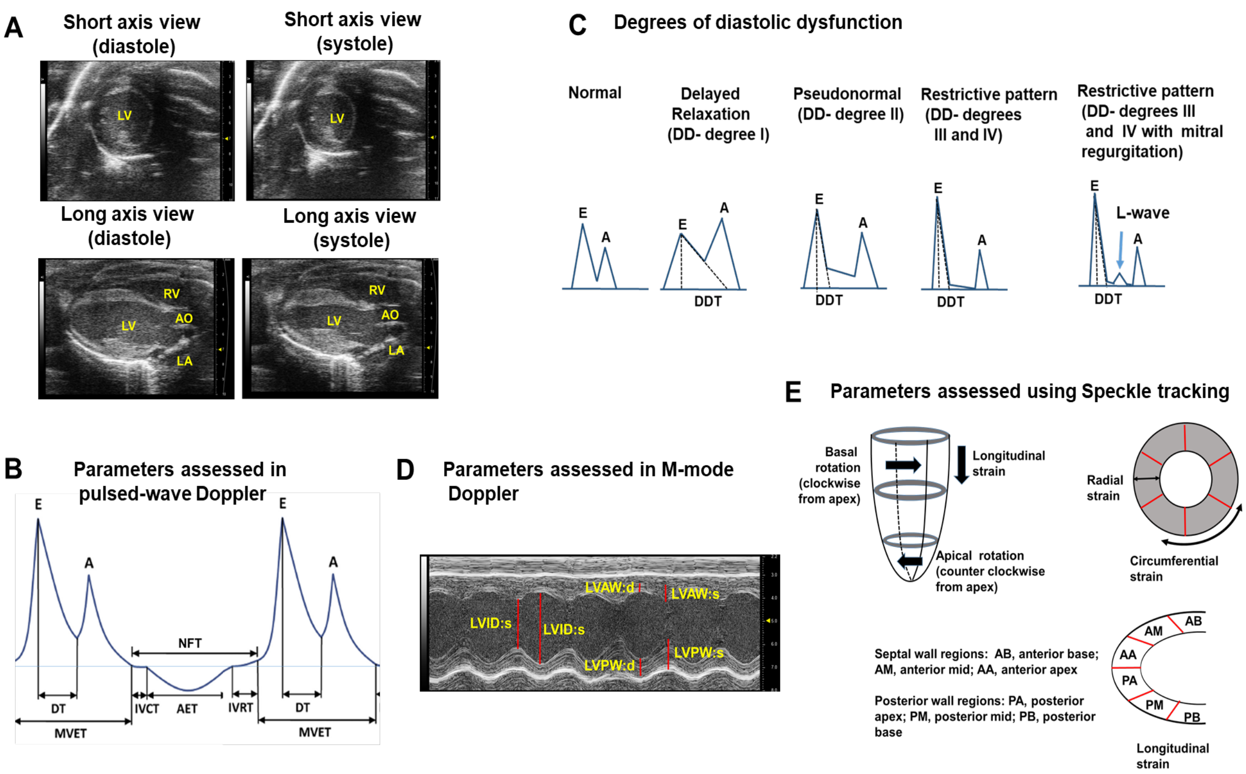

Supplemental Fig. 2: Short and long axes view of the heart and parameters assessed in conventional and Speckle tracking echocardiography. (A) Representative short axis (upper) and long axis (lower) echocardiographic views of the left ventricle of a mouse heart during diastole and systole. (B) Schematic of blood flow parameters measured using pulsed-wave Doppler echocardiography. (C) Schematic showing varying degrees of diastolic dysfunction including an L-wave. (D) Echocardiographic images showing contractile parameters measured using pulsed-wave Doppler echocardiography. (E) Schematic of strain parameters measured using speckle tracking echocardiography.

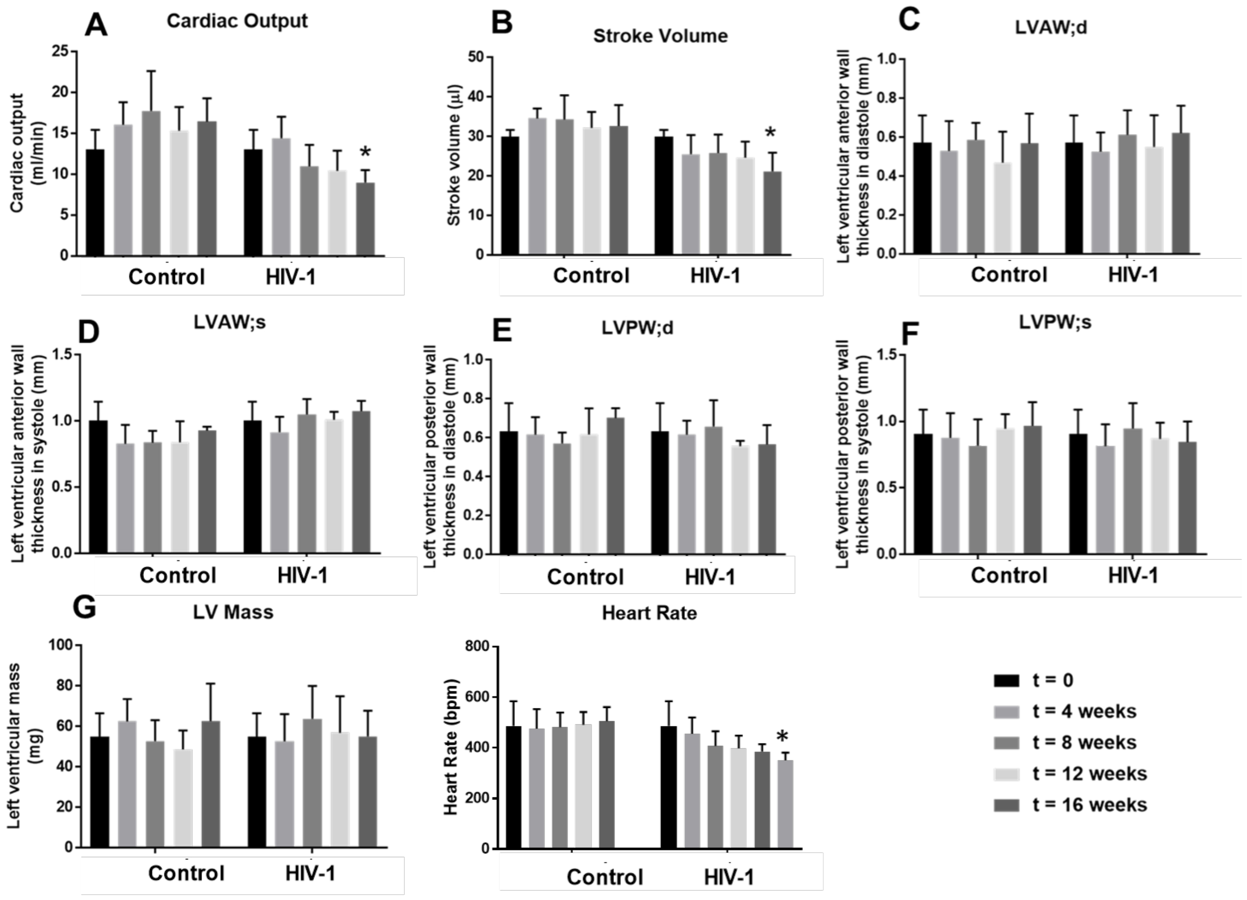

Supplemental Fig. 3: Longitudinal changes in systolic parameters and over a 16 week period after injecting Hu-NSG mice with saline or HIV-1 virus.

Panel A shows cardiac output; panel B, stroke volume; panel C, left ventricular anterior wall diameter at the end of diastole; panel D, left ventricular wall diameter at the end of systole; panel E, left ventricular posterior wall diameter at the end of diastole; panel F, left ventricular wall diameter at the end of systole; panel G, left ventricular mass and panel H, heart rate. Data shown are mean ± SEM for n = 8 uninfected Hu-NSG mice (3 males and 5 females) and n = 16 for HIV-1 infected Hu-NSG mice (4 males and 12 females). * denote significantly different from t = 0 weeks (p<0.05) within the group.

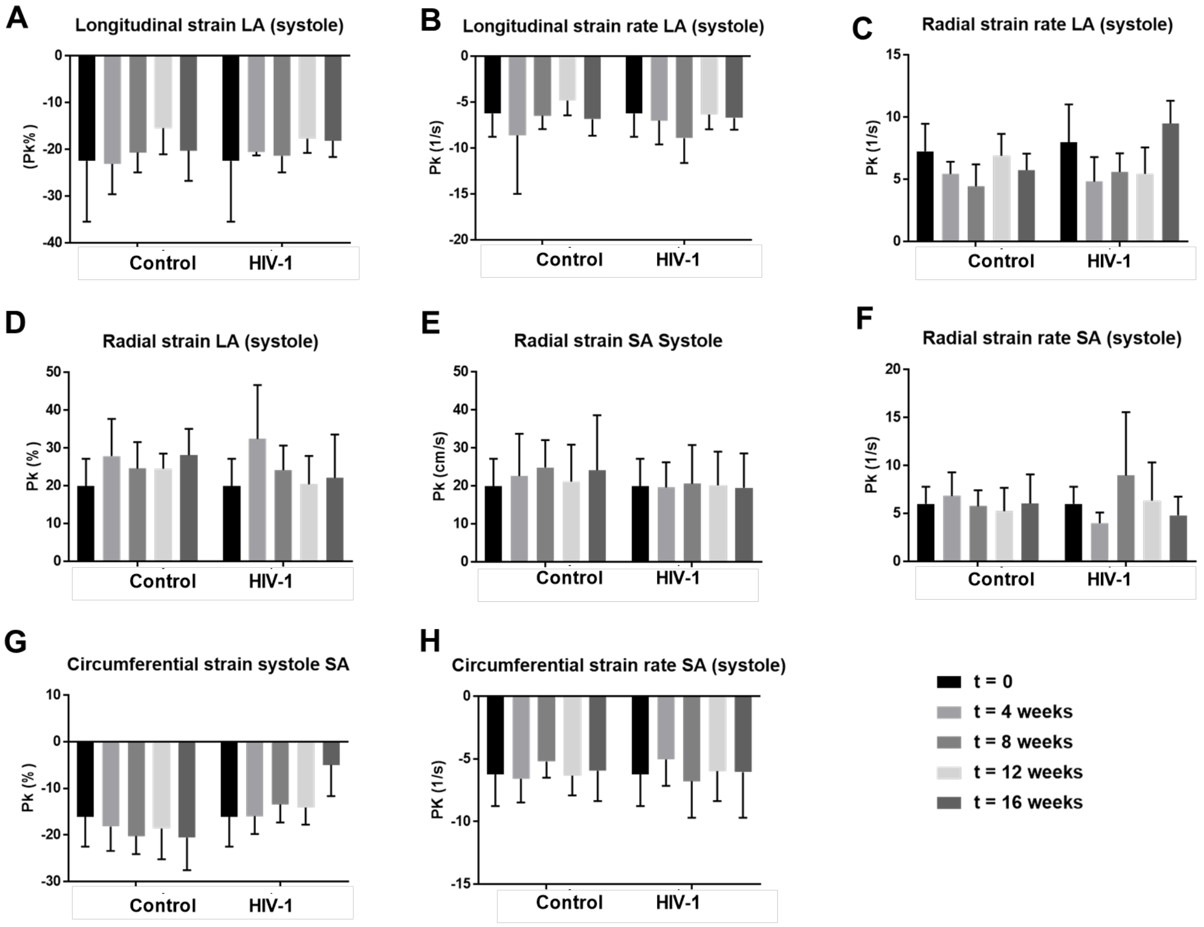

Supplemental Fig. 4: Longitudinal changes in strain parameters over a 16 week period after injecting Hu-NSG mice with saline or HIV-1 virus.

Panel A shows mean longitudinal strain in long axis mode; Panel B shows mean longitudinal strain rate in long axis mode; Panel C shows mean radial strain rate in long axis mode; Panel D shows mean radial strain in long axis mode; Panel E shows mean radial strain in short axis mode; Panel F shows mean radial strain rate in short axis mode; Panel G shows mean circumferential strain rate in short axis mode; Panels H shows mean circumferential strain rate in short axis mode Data shown are mean ± SEM for n = 8 uninfected Hu-NSG mice (3 males and 5 females) and n = 16 for HIV-1 infected Hu-NSG mice (4 males and 12 females). * denote significantly different from t = 0 weeks ($p<0.05$) within the group.

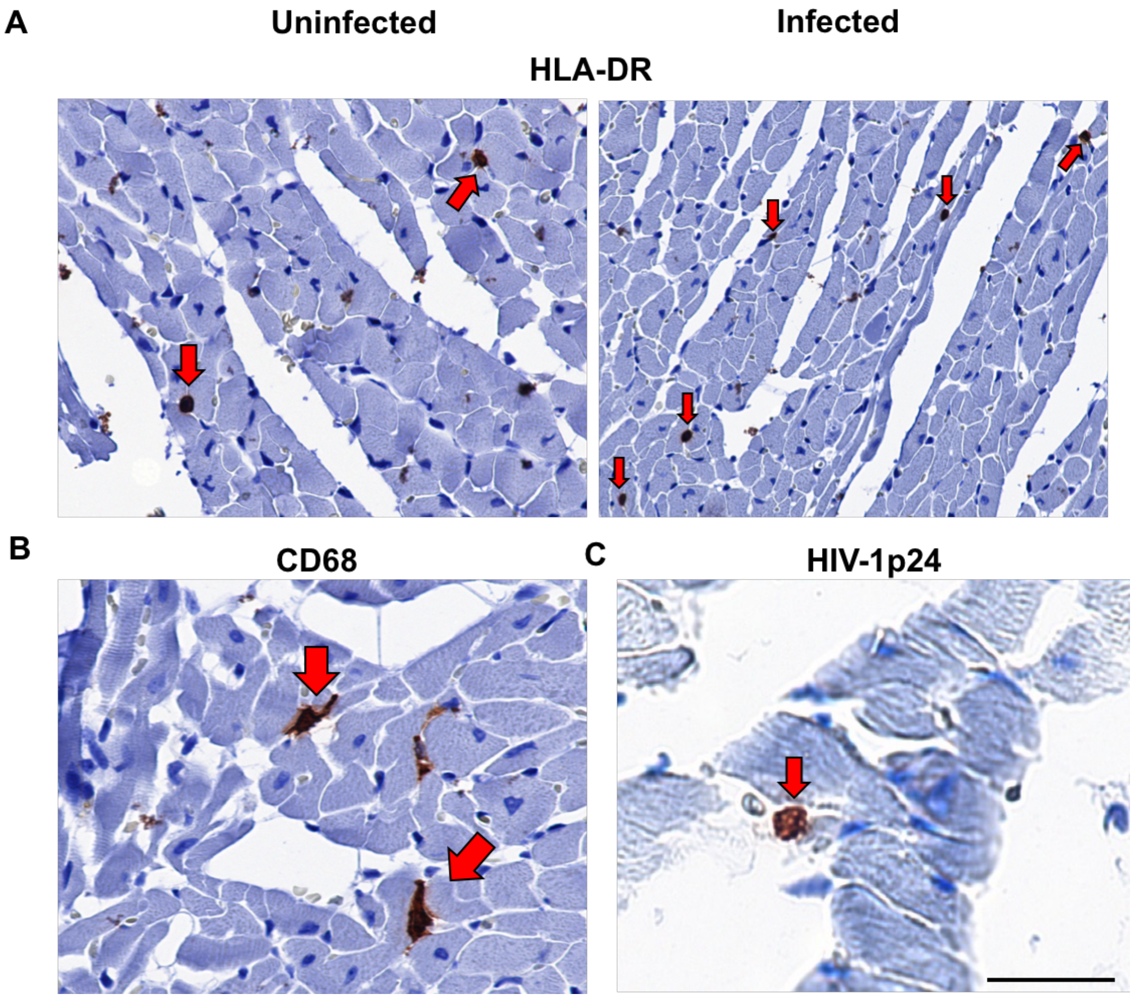

Supplemental Fig. 5: Immunohistological analysis of cardiac tissues of Humanized mice. (A) Representative heart regions in uninfected and HIV-1 infected animals. Paraffin sections (5μm) of heart were labelled with anti-HLA-DR to identify human activate cells (all positive labeling is brown). Sections were counter stained with hematoxylin. Images were captured at 20x. (**B**) Heart tissues were stained with anti-CD68 antibody to look for macrophages and the brown colored positive cells were shown by red arrows. (**C**) Presence of HIV-1 positive cells were observed by staining the tissues with anti-p24 antibodies to HIV-1. The images were captured at 40x magnification for B and C.

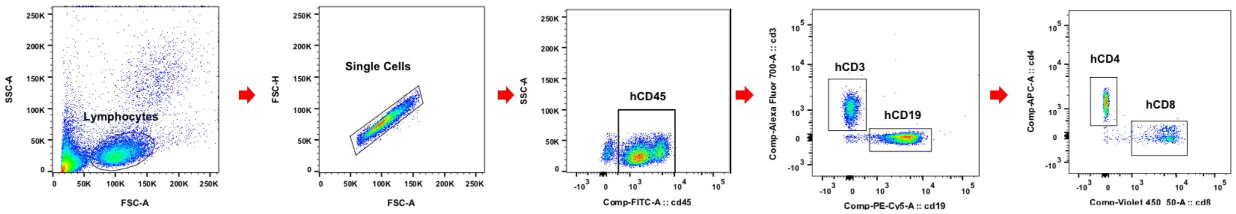

Supplemental Fig. 6: Flow cytometric gating strategy for human cell reconstitution in NSG-humanized mice. In brief, lymphocytes were first gated from whole blood cells by forward and side scatter analysis. Human cell (hCD45+) were then gated from total lymphocytes and single cells and subsequently grouped into human T lymphocytes (hCD3+) and B lymphocytes (hCD19+). Human CD3+ cells were then separated into human CD4+ (hCD4+) and CD8+ (hCD8+) T lymphocytes. This pattern of analysis is followed for all the time points pre- and post-HIV infection for immune cell profiling for each NSG-humanized mice.
